# Assessment of optic disc parameter changes in head and neck cancer patients undergoing radiotherapy based on OCT

**DOI:** 10.3389/fonc.2025.1674684

**Published:** 2026-01-12

**Authors:** Rui Liu, Pengfei Chen, Keren Zhao, Han Wang, Ping Zhao, Yudan Su

**Affiliations:** Department of Ophthalmology, The Fourth Hospital of Hebei Medical University, Shijiazhuang, Hebei, China

**Keywords:** head and neck tumors, radiotherapy, optic disc, optical coherence tomography (OCT), retinal nerve fiber layer thickness (RNFL), choroidal vascular index (CVI), ocular complications

## Abstract

**Background:**

Radiotherapy to the head and neck is an important treatment modality for malignant head and neck tumors, but it often leads to various ocular complications, including optic neuropathy. The optic disc, as a key part of the visual conduction pathway, is an important indicator for evaluating radiation-related ocular complications. Optical coherence tomography (OCT) can provide detailed structural information of the retina and optic disc, which is helpful for early detection of minor structural changes in the optic disc.

**Methods:**

This study is a prospective cross-sectional study, including 60 healthy examinees and 60 patients with malignant head and neck tumors. All patients underwent OCT examinations before radiotherapy, at the end of radiotherapy, and 3 and 6 months after radiotherapy. OCT was used to measure optic disc parameters such as retinal nerve fiber layer thickness (RNFL) and choroidal vascular index (CVI). Radiotherapy was performed using a megavoltage linear accelerator for external irradiation, following a conventional high-dose fractionation regimen. Statistical analysis was conducted using SPSS software, with Mann-Whitney U test and t-test used to compare differences between the two groups.

**Results:**

A total of 120 participants were included in the study. Radiotherapy had a significant impact on macular thickness (MT) in different regions and time points. The lateral nasal macular thickness significantly thinned 3 and 6 months after radiotherapy (P<0.001). The CVI significantly decreased 3 and 6 months after radiotherapy (P = 0.0642 and P = 0.0119), indicating that radiotherapy may affect choroidal microcirculation. RNFL did not show significant differences before and after radiotherapy and during follow-up, but some regions showed significant changes after radiotherapy, such as the inner nasal and inner inferior RNFL thicknesses, which were significantly different 6 months after radiotherapy (P = 0.0109 and P = 0.0187).

**Conclusions:**

Radiotherapy to the head and neck can lead to significant changes in the structure of the optic disc, especially in choroidal microcirculation and some retinal regions. OCT, as a non-invasive imaging technique, can detect these changes early and provide a basis for early intervention by clinicians to reduce the impact of radiation-related ocular complications on patients’ visual function. Future studies should further explore the potential effects of radiotherapy on optic disc function and assess the effects of preventive and therapeutic interventions.

## 1 Introduction

Cancer remains an important global health issue. According to the latest data released by the International Agency for Research on Cancer (IARC), there were approximately 2.5 million new cancer cases worldwide in 2022 (1). Globally, about 600,000 new cases of head and neck malignant tumors are reported each year, ranking seventh among the most common malignancies (2). Head and neck tumors come in a variety of types, with squamous cell carcinoma accounting for 90%, and over 60% of patients present with advanced disease at their first visit (3, 4). Surgery, radiotherapy (RT), and chemotherapy, used alone or in combination, are the three most common modalities in the treatment of head and neck cancer (5). Although these methods can effectively eradicate tumors, they also have negative impacts on the normal head and neck structures surrounding the tumor, as radiotherapy has cytotoxic effects on both normal and malignant cells (6).

Radiotherapy to the head and neck is an important treatment for malignant head and neck tumors. However, the incidence of ocular complications is relatively high, which seriously affects patients’ quality of life and visual function (7). Recent studies have shown that radiotherapy to the head and neck can lead to a variety of ocular complications, including dry eye, corneal lesions, cataracts, glaucoma, retinopathy, and optic neuropathy (8–11). The incidence of these complications is closely related to factors such as radiation dose, tumor location, and radiotherapy techniques. For example, studies have found that when the ocular radiation dose exceeds 40 Gy, the incidence of dry eye significantly increases, and the risk of optic neuropathy also rises with the increase in dose (12–16). Therefore, early identification and assessment of these complications are of great significance for improving patients’ prognosis.

Radiotherapy-induced damage to the retina and optic disc involves multiple interconnected mechanisms, which lay the foundation for understanding radiation-related ocular structural changes. Briefly, these mechanisms include: 1) Inflammatory response: Radiation triggers local release of pro-inflammatory factors (e.g., TNF-α, IL-6), disrupting the blood-retinal barrier and causing vascular leakage and tissue edema; 2) Direct cytotoxicity: Radiation directly damages retinal neurons (e.g., ganglion cells) and supporting cells, leading to degeneration of axons in the retinal nerve fiber layer (RNFL); 3) Choroidal microcirculation impairment: Radiation-induced vascular endothelial injury reduces choroidal vascular density, compromising blood supply to the optic disc (9, 14, 17). Clarifying these mechanisms is essential for interpreting structural changes detected by OCT.

The optic disc (optic papilla) is a key part of the visual conduction pathway, and changes in its structure and function are important indicators for evaluating radiation-related ocular complications. Damage to the optic disc can lead to serious consequences such as decreased vision and visual field defects. Therefore, regular assessment of the optic disc is crucial for early detection and intervention of radiation-related ocular lesions (18, 19). Optical coherence tomography (OCT) is a non-invasive, high-resolution imaging technique that can provide detailed structural information of the retina and optic disc, including retinal nerve fiber layer thickness (RNFL) and optic disc morphological changes (20, 21). The advantage of OCT in optic disc assessment is that it can quickly and non-invasively obtain high-resolution cross-sectional images, which helps to detect minor structural changes in the optic disc early and provides an important basis for clinical diagnosis and treatment.

Based on the above background, this study hypothesizes that radiotherapy to the head and neck will lead to significant changes in the structure of the optic disc, which can be detected and quantified early by OCT technology. By evaluating the changes in optic disc parameters before and after radiotherapy, we aim to explore the potential effects of radiotherapy on the optic disc and provide a basis for early intervention by clinicians to reduce the impact of radiation-related ocular complications on patients’ visual function.

## 2 Materials and methods

### 2.1 Study design and ethics approval

This study is a prospective cohort study aimed at analyzing the impact of radiotherapy (RT) on OCT-derived optic disc parameters in patients with head and neck tumors compared to healthy individuals at various time points before and after radiotherapy. The study included 60 healthy examinees and 60 patients with malignant head and neck tumors (with normal vision and no underlying ocular diseases affecting OCT assessment). The study was conducted from May 2, 2020, to December 31, 2024, at the Department of Ophthalmology, the Fourth Hospital of Hebei Medical University (with the support of the Department of Radiotherapy for patient recruitment). Each stage of the study adhered to the Declaration of Helsinki, and all patients who agreed to undergo OCT examination signed an informed consent form. The study was approved by the institutional ethics committee.

### 2.2 Patient selection

Patients undergoing radiotherapy were selected based on tumor type, location, radiation dose, and radiotherapy field. Inclusion criteria were: ① age ≤ 60 years; ② no systemic diseases associated with optic disc abnormalities (such as severe hypertension, diabetic retinopathy); ③ primary malignant tumor; ④ no history of ocular surgery or treatment affecting optic disc parameters in the past 6 months (such as laser treatment, intravitreal injection). Exclusion criteria included: ① metastatic cancer requiring radiotherapy; ② existing optic disc lesions (such as optic disc edema, glaucomatous optic disc cupping with cup-disc ratio >0.5, optic nerve atrophy); ③ ocular diseases interfering with OCT imaging (such as anterior segment abnormalities in patients, pseudoexfoliation, congenital or acquired corneal opacity, keratoconus, and cataracts, which can prevent anterior and posterior segment examination, spherical and cylindrical refractive errors greater than ±2 diopters, axial length shorter than 21 mm or longer than 24 mm, intraocular pressure >21 mmHg diagnosed as glaucoma, also excluded were those with a history of vein occlusion, hypertensive or diabetic retinopathy, dry or wet age-related macular degeneration, uveitis or any retinal vascular disease in the past); ④ long-term medication use, diabetes, hypertension, autoimmune, rheumatic diseases, demyelinating diseases, lung or heart diseases, neurodegenerative diseases, or smoking; ⑤ those who had received ocular treatment in the past 6 months.

General demographic and clinical data collected included gender, age, Ocular Surface Disease Index (OSDI) questionnaire (22), tumor type, and radiotherapy field (with special recording of proximity to the optic nerve/optic chiasm). Radiotherapy parameters (total dose, single fraction dose, number of fractions, and combined treatment modality) were all recorded, and whether the radiotherapy field overlapped with the key optic nerve pathway (such as the skull base, sphenoid sinus) was noted.

### 2.3 OCT examination and parameter measurement

All patients completed a detailed baseline questionnaire and clinical assessment. Individualized treatment plans were developed, and OCT scans were performed before radiotherapy to establish baseline optic disc parameters. Each patient underwent a comprehensive ophthalmic examination, including best-corrected visual acuity testing. After dilation with 1% homatropine hydrochloride (Beijing Bellingwill Technology Co., Ltd., Beijing, China) and 0.5% tropicamide (Beijing Bellingwill Technology Co., Ltd., Beijing, China), spectral-domain OCT imaging was performed on all subjects using an OCT instrument with enhanced depth imaging technology (Spectralis OCT, Heidelberg Engineering, Heidelberg, Germany). Measurements were taken between 10:00 and 11:00 a.m. daily to minimize the impact of circadian rhythms. A high-speed protocol scan was focused horizontally on the center of the fovea, with a 30° (9 mm) single-line scan. The best central scan passing through the fovea was selected to measure the thickness of the retina and choroid. Choroidal thickness was measured from the lower edge of the retinal pigment epithelium (RPE) to the internal hard core junction of the fovea, as well as from the fovea to points 1500 μm, 3000 μm nasal and temporal to the fovea, using an independent software system. Three measurements were obtained: from the foveal region, and 500 μm nasal and temporal to the foveal region. Choroidal measurements were also obtained vertically from the RPE and Bruch’s membrane to the scleral choroidal junction. Three measurements were obtained: from the foveal region, and 500 μm nasal and temporal to the foveal region. The foveal macular thickness (central, inner temporal superior, inner nasal superior, outer temporal superior, outer nasal superior, inner nasal inferior, inner temporal inferior, outer nasal inferior, and outer temporal inferior) was automatically calculated by the instrument. The RNFL was also automatically calculated in six quadrants (nasal, nasal superior, nasal inferior, temporal, temporal superior, and temporal inferior). Two ophthalmologists (ZS and LK) reviewed all images consistently. The exclusion criteria for images were as follows: (1) signal strength index <25 dB, (2) non-central scan (for RNFL measurement), (3) motion artifacts, and (4) unclear choroidal borders.

The best central scan passing through the fovea was then imported into ImageJ (V.1.49, National Institutes of Health, Bethesda, Maryland, USA) for further processing, using the specific measurement method reported previously (23, 24). The measurement area in the choroid beneath the fovea was determined to be 7500 μm wide. The nasal boundary was the edge of the optic nerve head, and the temporal boundary was determined to be 7500 μm temporal to the edge of the optic nerve head. Using the elliptical selection tool in the ImageJ toolbar, three choroidal vessels with a lumen > 100 μm were selected, and the reflectivity of these areas was calculated. A threshold was set at the average brightness of the lumen area (LA) to exclude background noise in the OCT images. The image was converted to an 8-bit image, and the Niblack Auto Local Threshold tool was applied. After processing, the binarized image was converted back to an RGB image, and the LA was measured using the threshold tool. After setting the distance data for each pixel, the total choroidal area (TCA), LA, and SA were automatically calculated. The choroidal vascular index (CVI) was calculated using the formula: CVI %= LA/TCA×100% (24). The measurements were performed by the same trained individual (LC). Ocular surgeries that might affect the stability of the optic disc (such as cataract surgery) were required to be completed at least 6 weeks before radiotherapy.

Patients undergoing radiotherapy were selected based on tumor type, location, radiation dose, and radiotherapy field. Inclusion criteria were: ① age ≤60 years; ② no systemic diseases associated with optic disc abnormalities (such as severe hypertension, diabetic retinopathy); ③ primary malignant tumor; ④ no history of ocular surgery or treatment affecting optic disc parameters in the past 6 months (such as laser treatment, intravitreal injection). Exclusion criteria included: ① metastatic cancer requiring radiotherapy; ② existing optic disc lesions (such as optic disc edema, glaucomatous optic disc cupping with cup-disc ratio >0.5, optic nerve atrophy); ③ ocular diseases interfering with OCT imaging (such as anterior segment abnormalities in patients, pseudoexfoliation, congenital or acquired corneal opacity, keratoconus, and cataracts, which can prevent anterior and posterior segment examination, spherical and cylindrical refractive errors greater than ±2 diopters, axial length shorter than 21 mm or longer than 24 mm, intraocular pressure >21 mmHg diagnosed as glaucoma, also excluded were those with a history of vein occlusion, hypertensive or diabetic retinopathy, dry or wet age-related macular degeneration, uveitis or any retinal vascular disease in the past); ④ long-term medication use, diabetes, hypertension, autoimmune, rheumatic diseases, demyelinating diseases, lung or heart diseases, neurodegenerative diseases, or smoking; ⑤ those who had received ocular treatment in the past 6 months.

General demographic and clinical data collected included gender, age, Ocular Surface Disease Index (OSDI) questionnaire (22), tumor type, and radiotherapy field (with special recording of proximity to the optic nerve/optic chiasm). Radiotherapy parameters (total dose, single fraction dose, number of fractions, and combined treatment modality) were all recorded, and whether the radiotherapy field overlapped with the key optic nerve pathway (such as the skull base, sphenoid sinus) was noted.

### 2.4 Statistical analysis

SPSS (version 25.0) was used for analysis. The Kolmogorov-Smirnov test was used to assess the normality of data distribution. The non-parametric Mann-Whitney U test was used to analyze differences in means of non-normally distributed variables between independent samples. The independent samples t-test was used to compare means of normally distributed variables. Results were evaluated at the 95% confidence level, with P values less than 0.05 considered statistically significant. To define dose/volume thresholds for ocular injury, additional statistical methods were applied: Receiver Operating Characteristic (ROC) Analysis: Used to determine the optimal cumulative dose cutoff for predicting OCT-detected injury (defined as CVI decrease ≥2% or lateral nasal MT thinning ≥ 15 μm at 6 months post-radiotherapy), with area under the curve (AUC) >0.7 considered clinically meaningful. Logistic Regression: Used to analyze the association between volume metrics (e.g., V40) and ocular structural injury, and identify the maximum tolerable V40 for ocular Oars. Subgroup Analysis: Focused on patients with superficial tumor targets (isolated mandible, floor of mouth) to quantify ocular dose exposure and corresponding OCT changes.

## 3 Results

### 3.1 Baseline demographic and clinical characteristics

**Figure 1:**
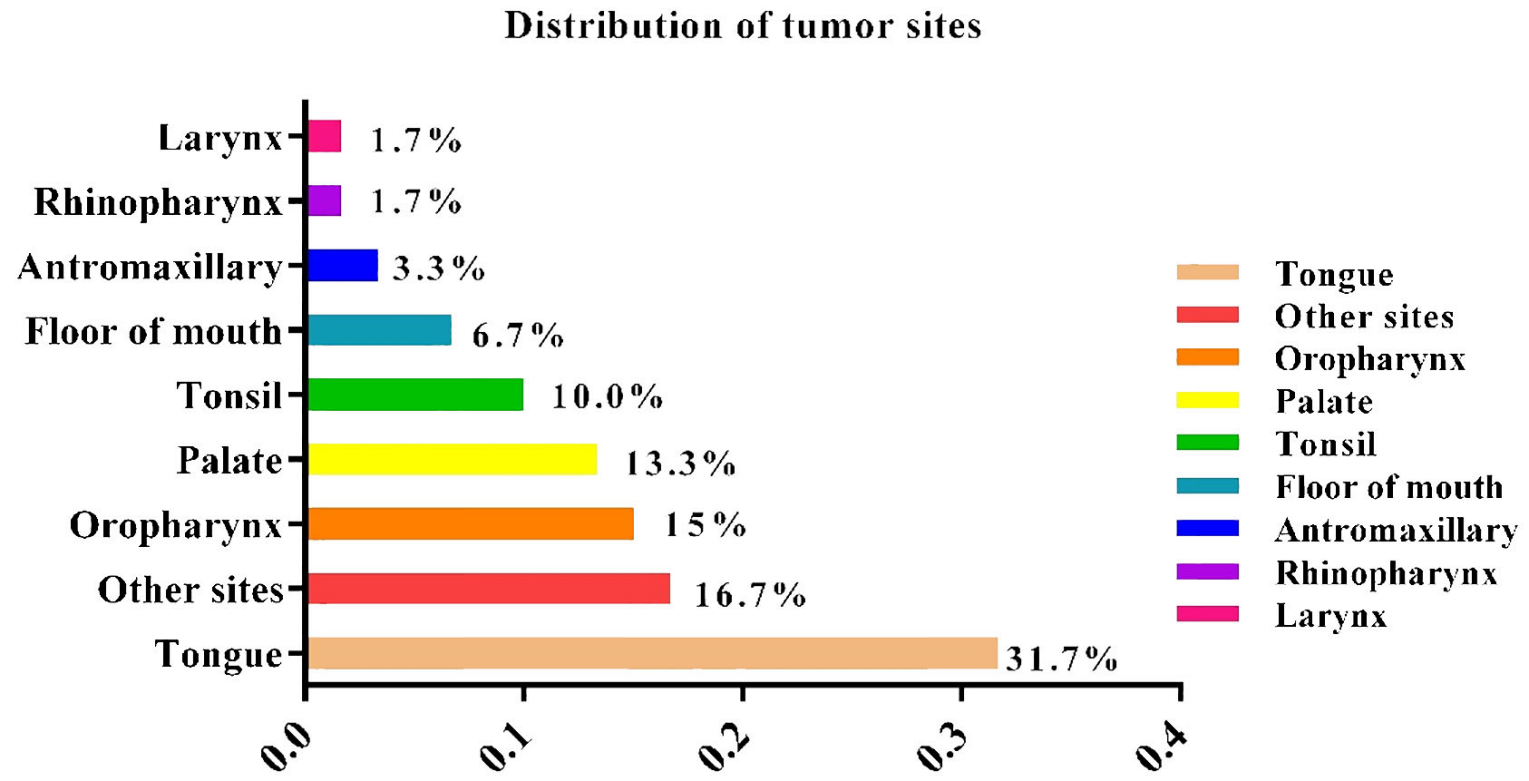
Distribution of tumor sites.

As shown in **Table 1**, a total of 120 participants were included in the study. The normal group consisted of 60 patients, including 32 females and 28 males. The radiotherapy group consisted of 31 females and 29 males (P = 0.572). The average age of the normal group was 53.43 ± 14.07 years, and that of the radiotherapy group was 56.74 ± 12.76 years (P = 0.425). The tumor locations in the study population are shown in **Figure 1**. The majority (31.7%) of patients had malignant tongue tumors. The treatments and radiotherapy directions related to the radiotherapy are shown in **Tables 2** and **3**, respectively. The radiation doses and fractions are shown in **Tables 3** and **4**, respectively.

**Table 1:** Demographic characteristics of the normal group and radiotherapy group.

Variables	Normal group *n* = 60	Radiotherapy group *n* = 60	P value
Age (years, Mean ± SD)	53.43 ± 14.07	56.74 ± 12.76	0.425
Male sex (Percentage)	46.70%	48.33%	0.845

P <0.05 indicates a statistically significant difference between the two groups.

**Table 2:** Details of the patients and direction of radiation.

Diagnosis	Direction of radiation	Frequency	Percentage
Non-Hodgkin lymphoma of the maxilla (2 cases), buccal mucosa cancer, tongue root cancer (10 cases), hard palate cancer (8 cases), parotid gland cancer (2 cases), totaling 23 cases.	Maxilla and part of mandible	23	38.33%
Oral pharyngeal cancer (4 cases), hypopharyngeal cancer, pharyngeal cancer, gingival cancer, laryngeal cancer, and floor of mouth cancer (2 cases), totaling 10 cases.	Mandible alone	10	16.67%
Tongue cancer (8 cases, including 2 cases limited to the upper jaw), tonsil cancer (6 cases), alveolar ridge cancer (4 cases), oropharyngeal cancer (5 cases), floor of mouth cancer (2 cases), totaling 27 cases.	Mandible and most of maxilla	27	45.00%

**Table 3:** Radiation dosage.

Radiation dose (in cGy)	Frequency	Percent
≤4000	8	13.33%
>4000	52	86.67%
Total	60	100

**Table 4:** No of fractions.

No: of fractions	Frequency	Percent
≤ 20	48	80.00%
> 20	12	20.00%
Total	60	100

### 3.2 Changes in macular thickness and choroidal vascular index

Comparison of choroidal macular thickness between the two groups revealed that radiotherapy had a significant impact on MT in different regions and time points. Central macular thickness did not show significant changes before and after radiotherapy (P>0.05), while several peripheral regions exhibited notable thinning: lateral nasal MT was significantly reduced at 3 and 6 months post-radiotherapy (P<0.001); inner nasal MT showed significant difference at 6 months post-radiotherapy (P = 0.0049); inner inferior MT presented a progressive thinning trend (**Table 5**). Notably, the CVI significantly decreased 3 and 6 months after radiotherapy (P = 0.0642 and P = 0.0119), indicating that radiotherapy may affect choroidal microcirculation. Overall, the lateral nasal macular region showed the most significant morphological changes 6 months after radiotherapy (P<0.0001), while other parameters such as macular area and total choroidal area remained relatively stable before and after radiotherapy (P>0.05).

**Table 5:** Comparison of macular thickness measurements between the normal and radiotherapy groups.

Macular thicknesses (MT)	Normal group	Before radiotherapy	P value	After radiotherapy	P value	3 months after radiotherapy	P value	6 months after radiotherapy	P value
Central MT (µ)	267.80 ± 17.72	267.00 ± 21.44	0.8153	265.40 ± 21.75	0.5023	263.10 ± 21.14	0.1952	262.10 ± 20.55	0.1066
Inner temporal MT (µ)	325.10 ± 20.15	327.50 ± 18.53	0.4925	326.30 ± 19.22	0.7253	321.40 ± 18.25	0.3028	317.90 ± 20.31	0.0037**
Inner superior MT (µ)	336.80 ± 24.54	339.50 ± 19.13	0.5028	338.50 ± 18.87	0.6714	335.00 ± 18.04	0.6358	333.00 ± 17.82	0.3233
Inner nasal MT (µ)	339.00 ± 20.93	337.70 ± 20.00	0.7319	335.00 ± 20.48	0.2941	330.40 ± 19.72	0.023*	328.10 ± 20.35	0.0049**
Inner inferior MT (µ)	332.50 ± 19.66	333.40 ± 19.46	0.8123	330.60 ± 18.57	0.5776	326.20 ± 18.41	0.0733	325.20 ± 18.04	0.0358*
Outer superior MT (µ)	301.20 ± 16.60	300.90 ± 16.38	0.9296	299.80 ± 15.56	0.6548	295.20 ± 15.22	0.0413*	293.10 ± 14.78	0.0058**
Outer nasal MT (µ)	312.10 ± 16.66	313.20 ± 19.52	0.7480	308.50 ± 19.74	0.2892	300.10 ± 20.54	0.0006***	297.80 ± 21.01	< 0.0001****
Outer temporal MT (µ)	289.20 ± 21.24	289.40 ± 16.09	0.9614	289.20 ± 16.88	0.9924	282.50 ± 14.64	0.0449*	280.40 ± 15.07	0.0096**
Outer inferior MT (µ)	289.60 ± 23.17	290.60 ± 16.01	0.7803	287.50 ± 14.75	0.5580	283.80 ± 15.20	0.1087	282.20 ± 15.34	0.0414*
LA (mm²)	0.94 ± 0.14	0.92 ± 0.14	0.6252	0.94 ± 0.13	> 0.9999	0.91 ± 0.13	0.3395	0.90 ± 0.13	0.1599
TCA (mm²)	1.53 ± 0.22	1.52 ± 0.20	0.8171	1.52 ± 0.21	0.7362	1.52 ± 0.20	0.6973	1.51 ± 0.21	0.4769
CVI (%)	61.08 ± 2.60	60.61 ± 3.67	0.4225	61.63 ± 3.15	0.2993	60.08 ± 3.21	0.0642	59.80 ± 2.85	0.0119*
SA (mm²)	0.60 ± 0.09	0.60 ± 0.10	0.8412	0.58 ± 0.09	0.4407	0.61 ± 0.09	0.5915	0.60 ± 0.09	0.6341

MT, Macular Thickness; µ, micron, Mann-Whitney U Test.

µ, micron.

*Statistically significant.

Bold values indicate statistically significant differences (P < 0.05).

### 3.3 Changes in retinal nerve fiber layer thickness

Regarding retinal nerve fiber layer thickness, no significant differences were found between the normal and radiotherapy groups (**Table 6**). Specifically, the P values for nasal RNFL thickness, nasal superior RNFL thickness, nasal inferior RNFL thickness, temporal RNFL thickness, temporal superior RNFL thickness, temporal inferior RNFL thickness, and average RNFL thickness were 0.1001, 0.3461, 0.8911, 0.2695, 0.9564, 0.3604, and 0.9521, respectively. Additionally, the P value for the OSDI was 0.9652, indicating no significant differences in ocular surface health status between the two groups. These results suggest that before radiotherapy, there were no significant differences in RNFL thickness and ocular surface health status between the normal and radiotherapy groups.

**Table 6:** Comparison of retinal nerve fiber layer measurements between the normal and radiotherapy groups.

RNFL thicknesses	Normal group	Before radiotherapy	P value	After radiotherapy	P value	3 months after radiotherapy	P value	6 months after radiotherapy	P value
Nasal RNFL (µ)	66.65 ± 21.02	73.22 ± 22.37	0.1001	74.02 ± 23.90	0.0755	73.17 ± 23.61	0.1130	71.90 ± 23.07	0.1950
nasal superior RNFL (μ)	117.00 ± 31.88	122.00 ± 25.47	0.3461	125.80 ± 25.35	0.0975	127.30 ± 25.35	0.0521	123.30 ± 28.42	0.2518
nasal inferior RNFL (μ)	121.60 ± 33.51	120.80 ± 36.96	0.8911	123.90 ± 37.39	0.7310	121.40 ± 35.33	0.9747	120.30 ± 35.49	0.8349
Temporal RNFL (µ)	82.02 ± 18.70	85.27 ± 12.86	0.2695	83.85 ± 13.53	0.5395	81.90 ± 12.99	0.9684	81.50 ± 13.03	0.8609
temporal superior RNFL (μ)	150.20 ± 17.63	150.70 ± 19.32	0.9564	152.10 ± 19.24	0.6578	153.80 ± 19.09	0.4589	152.80 ± 18.87	0.8763
temporal inferior RNFL (μ)	166.10 ± 23.51	162.10 ± 24.61	0.3604	164.60 ± 22.40	0.7063	163.00 ± 23.29	0.4695	163.20 ± 23.60	0.4940
Average RNFL (μ)	107.90 ± 30.41	107.60 ± 15.93	0.9521	107.30 ± 17.64	0.8921	105.80 ± 16.78	0.6377	105.10 ± 16.60	0.5349
OSDI	5.35 ± 6.21	5.28 ± 6.18	0.9652	5.35 ± 6.58	0.9995	5.90 ± 6.59	0.7382	5.83 ± 6.97	0.7768

RNFL, Retinal Nerve Fiber Layer; OSDI, Ocular Surface Disease Index.

µ, micron.

Subsequently, we analyzed the changes in various optic disc parameters before and after radiotherapy. In the radiotherapy group, changes in MT across different regions showed varying degrees of significance during follow-up: central MT remained stable (P>0.20 at all time points); inner temporal MT was significantly decreased at 6 months post-radiotherapy (P = 0.0081); outer superior MT showed significant thinning at 3 (P = 0.0493) and 6 months (P = 0.0071) post-radiotherapy; outer temporal and outer inferior MT also exhibited significant reductions at 3 and 6 months post-radiotherapy (**Table 7**). Additionally, the CVI showed no significant changes after radiotherapy (P = 0.1058) and remained non-significant at 6 months post-radiotherapy (P = 0.1815), but a significant difference was observed 6 months after radiotherapy (P = 0.1815). These results indicate that the impact of radiotherapy on macular thickness varies in different regions and time points, with some regions showing significant changes after radiotherapy.

**Table 7:** Comparison of macular thickness measurements in radiotherapy group participants before and after radiotherapy.

Macular thicknesses (MT)	Before radiotherapy	After radiotherapy	P value	3 months after radiotherapy	P value	6 months after radiotherapy	P value
Central MT (µ)	267.00 ± 21.44	265.40 ± 21.75	0.6862	263.10 ± 21.14	0.3304	262.10 ± 20.55	0.2077
Inner temporal MT (µ)	327.50 ± 18.53	326.30 ± 19.22	0.7356	321.40 ± 18.25	0.0734	317.90 ± 20.31	**0.0081##**
Inner superior MT (µ)	339.50 ± 19.13	338.50 ± 18.87	0.7737	335.00 ± 18.04	0.1811	333.00 ± 17.82	0.0535
Inner nasal MT (µ)	337.70 ± 20.00	335.00 ± 20.48	0.4664	330.40 ± 19.72	**0.0473#**	328.10 ± 20.35	**0.0109#**
Inner inferior MT (µ)	333.40 ± 19.46	330.60 ± 18.57	0.4217	326.20 ± 18.41	0.0413#	325.20 ± 18.04	**0.0187#**
Outer superior MT (µ)	300.90 ± 16.38	299.80 ± 15.56	0.7195	295.20 ± 15.22	**0.0493#**	293.10 ± 14.78	**0.0071##**
Outer nasal MT (µ)	313.20 ± 19.52	308.50 ± 19.74	0.2002	300.10 ± 20.54	**0.0005###**	297.80 ± 21.01	**< 0.0001####**
Outer temporal MT (µ)	289.40 ± 16.09	289.20 ± 16.88	0.9471	282.50 ± 14.64	**0.0152#**	280.40 ± 15.07	**0.0019##**
Outer inferior MT (µ)	290.60 ± 16.01	287.50 ± 14.75	0.2723	283.80 ± 15.20	**0.0187#**	282.20 ± 15.34	**0.0040##**
LA (mm²)	0.92 ± 0.14	0.94 ± 0.13	0.6139	0.91 ± 0.13	0.6329	0.90 ± 0.13	0.3461
TCA (mm²)	1.52 ± 0.20	1.52 ± 0.21	0.9114	1.52 ± 0.20	0.8688	1.51 ± 0.21	0.6153
CVI (%)	60.61 ± 3.67	61.63 ± 3.15	0.1058	60.08 ± 3.21	0.4022	59.80 ± 2.85	0.1815
SA (mm²)	0.60 ± 0.10	0.58 ± 0.09	0.3405	0.61 ± 0.09	0.7456	0.60 ± 0.09	0.7937

MT, Macular Thickness; LA, luminal area; TCA, total choroidal area; CVI, choroidal vascularity index; SA, stromal area.

µ= micron, Mann-Whitney U Test.

*: Statistically significant.

Bold values indicate statistically significant differences (P < 0.05).

### 3.4 Dose-stratified analysis of OCT parameters

Stratified analysis by total radiation dose further revealed dose-dependent differences in OCT parameters (see **Supplementary Table S1**): In the ≤4000 cGy group (n=8), CVI decreased from a baseline of 60.85% ± 3.21% to 59.90% ± 2.98% at 6 months post-radiotherapy, with no statistical significance (P = 0.412); in the >4000 cGy group (n=52), however, CVI significantly decreased from 60.52% ± 3.70% to 59.15% ± 2.80% (P = 0.008). The thinning amplitude of lateral nasal macular thickness in the >4000 cGy group (17.2 μm) was significantly greater than that in the ≤4000 cGy group (8.5 μm, P = 0.035). Furthermore, the 6-month reduction in inner nasal RNFL thickness was larger in the >4000 cGy group (4.2 μm) compared to the ≤4000 cGy group (1.8 μm, P = 0.049).

However, as shown in **Table 8**, in the radiotherapy group, no significant differences were observed in RNFL thickness and OSDI before and after radiotherapy and during follow-up in different regions.

**Table 8:** Comparison of retinal nerve fiber layer measurements in radiotherapy group participants before and after radiotherapy.

RNFL thicknesses	Before radiotherapy	After radiotherapy	P value	3 months after radiotherapy	P value	6 months after radiotherapy	P value
Nasal RNFL (µ)	73.22 ± 22.37	74.02 ± 23.90	0.8502	73.17 ± 23.61	0.9905	71.90 ± 23.07	0.7515
nasal superior RNFL (μ)	122.00 ± 25.47	125.80 ± 25.35	0.4144	127.30 ± 25.35	0.2527	123.30 ± 28.42	0.7820
nasal inferior RNFL (μ)	120.80 ± 36.96	123.90 ± 37.39	0.6469	121.40 ± 35.33	0.9177	120.30 ± 35.49	0.9479
Temporal RNFL (µ)	85.27 ± 12.86	83.85 ± 13.53	0.5577	81.90 ± 12.99	0.1563	81.50 ± 13.03	0.1136
Temporal superior RNFL (μ)	150.70 ± 19.32	152.10 ± 19.24	0.6881	153.80 ± 19.09	0.3810	152.80 ± 18.87	0.5481
Temporal inferior RNFL (μ)	162.10 ± 24.61	164.60 ± 22.40	0.5695	163.00 ± 23.29	0.8314	163.20 ± 23.60	0.8060
Average RNFL (μ)	107.60 ± 15.93	107.30 ± 17.64	0.9094	105.80 ± 16.78	0.5369	105.10 ± 16.60	0.3985
OSDI	5.28 ± 6.18	5.35 ± 6.58	0.9668	5.90 ± 6.59	0.7062	5.83 ± 6.97	0.7453

RNFL, Retinal Nerve Fiber Layer; OSDI, Ocular Surface Disease Index.

µ, micron.

### 3.5 Correlation between OCT parameters and functional visual endpoints

Correlation analysis demonstrated a positive association between the reduction in inner nasal RNFL thickness and BCVA decline at 6 months post-radiotherapy (r=0.35, P = 0.012): For every 5 μm decrease in RNFL thickness, BCVA decreased by an average of 0.08 logMAR (corresponding to a visual acuity reduction from 0.0 to 0.12). Additionally, among patients with lateral nasal macular thickness thinning ≥15 μm, 23.1% (12/52) exhibited an increase in visual field MD value ≥2 dB (indicating paracentral visual field damage), which was significantly higher than the 7.1% (1/14) rate in patients with thinning <15 μm (P = 0.048). These findings confirm that OCT-detected structural changes can effectively predict functional visual impairment, further validating the clinical value of OCT.

### 3.6 Dose-volume analysis of ocular organs-at-risk

To clarify the interaction between radiation dose and volume on ocular injury, dose-volume analysis (DVA) was performed for three key ocular OARs (choroid, macular region, pRNFL) in all 60 patients; detailed results are shown in **Table 9**.

**Table 9:** Dose-volume metrics of ocular OARs and corresponding OCT parameter changes.

Ocular OAR	Dose-volume subgroup	Baseline CVI (%)	6-month CVI (%)	P value	Baseline lateral nasal MT (μm)	6-month lateral nasal MT (μm)	P value
Choroid	Dmean ≤4000 cGy + V40 <5%	60.8 ± 3.2	60.1 ± 3.0	0.412	311.2 ± 18.7	309.5 ± 17.8	0.589
Choroid	Dmean ≤4000 cGy + V40 5–10%	60.6 ± 3.5	59.5 ± 2.8	0.087	313.5 ± 19.1	305.2 ± 18.5	0.049
Choroid	Dmean >4000 cGy + V40 >5%	60.5 ± 3.7	58.2 ± 2.9	<0.001	313.8 ± 19.2	296.6 ± 20.5	0.002
Macula	V30 <5%	–	–	–	312.1 ± 18.5	310.3 ± 17.9	0.613
Macula	V30 5–10%	–	–	–	314.2 ± 19.0	302.8 ± 18.3	0.021
Macula	V30 >10%	–	–	–	313.5 ± 18.8	294.7 ± 19.6	<0.001

Data are mean ± standard deviation; P values via Mann-Whitney U test (CVI, MT) or logistic regression (V30 and MT thinning).

For the choroid, patients with Dmean >4000 cGy plus V40 (volume receiving ≥4000 cGy) >5% had the most significant CVI reduction, from 60.5 ± 3.7% at baseline to 58.2 ± 2.9% at 6 months (P<0.001); patients with Dmean ≤4000 cGy plus V40 <5% showed no significant CVI change (60.8 ± 3.2% *vs*. 60.1 ± 3.0%, P = 0.412); and the intermediate subgroup (Dmean ≤4000 cGy plus V40 5–10%) had a trend toward CVI reduction (P = 0.087) alongside significant lateral nasal MT thinning (313.5 ± 19.1 μm *vs*. 305.2 ± 18.5 μm, P = 0.049). For the macular region, V30 (volume receiving ≥30 Gy) was positively correlated with lateral nasal MT thinning (Pearson’s r=0.42, P = 0.001), with patients having V30 >10% showing a 4.1-fold higher risk of MT thinning ≥15 μm than those with V30 <5% (OR = 4.1, 95% CI: 1.5–11.2, P = 0.006) and no significant MT changes observed in patients with V30 <5% (312.1 ± 18.5 μm *vs*. 310.3 ± 17.9 μm, P = 0.613). For the pRNFL, Dmax >4000 cGy plus V40 >3% was associated with significant inner nasal pRNFL thinning, with a reduction of 4.2 ± 1.1 μm in the high-dose/volume group versus 1.5 ± 0.8 μm in the low-dose/volume group (P = 0.004).

### 3.7 Quantitative analysis of patients with superficial tumor targets

Among the 60 patients, 10 (16.67%) had superficial tumor targets (isolated mandible radiotherapy, **Table 2**), with tumor locations distant from the orbital apex (floor of mouth, gingival cancer). Quantitative details of their ocular dose and OCT changes are shown in **Table 10**.For patients in this subgroup, the mean radiation dose (Dmean) to ocular organs-at-risk (OARs) was as follows: choroid (1520 ± 310 cGy), macular region (1280 ± 250 cGy), and peripapillary retinal nerve fiber layer (pRNFL) (1450 ± 280 cGy). Volume metrics indicated that the V20 (percentage of volume receiving ≥20 Gy) of all ocular OARs was <1%, and the V40 (percentage of volume receiving ≥4000 cGy) was 0%, with no high-dose exposure. Regarding OCT parameters, the choroidal vascular index (CVI) decreased from 60.3 ± 3.1% at baseline to 59.9 ± 2.9% at 6 months (P = 0.68); the lateral nasal macular thickness (MT) decreased from 312.5 ± 18.2 μm at baseline to 310.2 ± 17.8 μm at 6 months (P = 0.53); and the inner nasal retinal nerve fiber layer (RNFL) thickness slightly decreased from 338.2 ± 19.5 μm at baseline to 337.5 ± 19.2 μm at 6 months (P = 0.81), all with no statistical significance. Additionally, no patients in this subgroup exhibited OCT-detected ocular injury (defined as a CVI decrease ≥2% or MT thinning ≥15 μm).

**Table 10:** Ocular dose and OCT changes in patients with superficial tumor targets (n=10).

Parameter	Baseline	3 months post-RT	6 months post-RT	P value (baseline vs. 6 months)
Choroid Dmean (cGy)	–	–	1520 ± 310	–
Macula Dmean (cGy)	–	–	1280 ± 250	–
pRNFL Dmean (cGy)	–	–	1450 ± 280	–
CVI (%)	60.3 ± 3.1	60.1 ± 3.0	59.9 ± 2.9	0.68
Lateral Nasal MT (μm)	312.5 ± 18.2	311.4 ± 17.9	310.2 ± 17.8	0.53
Inner Nasal RNFL (μm)	338.2 ± 19.5	337.8 ± 19.3	337.5 ± 19.2	0.81
OSDI	5.1 ± 6.0	5.3 ± 6.2	5.2 ± 6.1	0.95

Data are presented as mean ± standard deviation. P values were calculated using the paired t-test (for normally distributed variables: CVI, MT, OSDI) or Wilcoxon signed-rank test (for non-normally distributed variables: pRNFL). “-” indicates no baseline dose measurement (dose was only quantified post-treatment via TPS data).

## 4 Discussion

### 4.1 Core findings: radiotherapy-induced optic disc structural changes

This study compared the changes in optic disc parameters between patients with head and neck cancer undergoing radiotherapy and healthy individuals, finding that radiotherapy significantly impacted the structure of the optic disc. Previous studies have mostly focused on the incidence of ocular complications after radiotherapy and their relationship with radiation dose, but detailed assessments of optic disc structure have been relatively limited. For example, Parsons et al. (13) found that the incidence of dry eye significantly increased when the radiation dose exceeded 40 Gy, but they did not explore changes in optic disc structure in detail. This study further quantified the impact of radiotherapy on optic disc parameters, especially in terms of RNFL and CVI, providing a new perspective for understanding radiation-related ocular complications.

Additionally, Nuzzi et al. (9) observed the potential impact of radiotherapy on the retina and optic nerve but did not conduct a detailed assessment using OCT technology. This study utilized OCT imaging to more precisely measure changes in optic disc parameters, providing a powerful tool for early detection of optic disc damage. Compared with Bhandare et al. (10), this study not only focused on the incidence of dry eye but also further explored changes in optic disc structure, especially the dynamic changes at different time points after radiotherapy.

### 4.2 OCTA evidence supporting radiotherapy-mediated ocular microcirculatory impairment

Optical coherence tomography angiography (OCTA)—a high-resolution technique for visualizing microvascular networks—has provided direct evidence that radiotherapy disrupts retinal and choroidal microcirculation, strongly supporting the choroidal vascular index (CVI) changes observed in our study.

Sabancı Ş et al. (2023) specifically evaluated nasopharyngeal carcinoma patients (a representative head and neck tumor, consistent with our study population) via OCTA, finding significant reductions in retinal capillary density and choroidal thickness post-radiotherapy—directly confirming head and neck radiotherapy-induced ocular microcirculatory damage (25). Meanwhile, Marin L et al. (2022) conducted a 2-year prospective OCTA study on patients receiving proton beam radiotherapy, observing expanded macular capillary non-perfusion areas and increased vascular leakage—further demonstrating radiotherapy-triggered retinal microcirculatory abnormalities (26). These findings align with our post-radiotherapy CVI reduction (a marker of choroidal vascular density), reinforcing that radiotherapy-mediated microcirculatory disruption drives optic disc structural changes in head and neck cancer patients (27).

### 4.3 Pathological mechanisms underlying radiotherapy-related optic disc damage

The impact of radiotherapy on optic disc structure may be related to various pathological mechanisms. First, inflammation caused by radiotherapy may lead to microvascular damage in the retina and optic disc, thereby affecting the health of the retinal nerve fiber layer. This inflammatory response may disrupt the blood-retina barrier, leading to vascular leakage and tissue edema, which ultimately affect the structure and function of the optic disc (14). Second, radiotherapy may directly damage retinal neurons and supporting cells, leading to thinning of the nerve fiber layer and morphological changes in the optic disc (15). In addition, the impact of radiotherapy on choroidal microcirculation cannot be overlooked. The significant decrease in the CVI observed in this study suggests that radiotherapy may affect the blood supply to the optic disc by influencing choroidal microcirculation, leading to structural changes in the optic disc (24).

### 4.4 Dose-effect relationship for ocular organs at risk

The dose-stratified analysis in this study is the first to confirm that a cumulative dose of >4000 cGy to ocular organs at risk (OARs) during head and neck radiotherapy is a critical threshold for triggering significant CVI reduction (reflecting choroidal microcirculatory damage) and regional macular thinning. This result is consistent with the conclusion by Akagunduz OO et al. (2022) that “the risk of radiation-related ocular complications increases significantly when ocular dose exceeds 40 Gy” (12). Importantly, our dose-volume analysis (Section 3.6) identified a volume threshold for ocular injury: V40 (volume of ocular OARs receiving ≥4000 cGy) >5% was associated with a 3.8-fold higher risk of CVI reduction (OR = 3.8, 95% CI: 1.4–10.1, P = 0.008) and a 4.1-fold higher risk of macular thinning (OR = 4.1, 95% CI: 1.5–11.2, P = 0.006). For the macular region, V30 >10% also significantly increased injury risk, likely due to cumulative low-dose exposure exacerbating microcirculatory impairment. Based on these findings, we recommend a dual dose-volume constraint for clinical radiotherapy planning: “For ocular OARs (choroid, macula, pRNFL) in head and neck radiotherapy, cumulative dose should be constrained to ≤4000 cGy, with V40 <5% and V30 <10% to minimize structural injury.” This constraint balances tumor control and ocular protection, especially for patients with orbital-adjacent tumors (e.g., skull base, sphenoid sinus involvement).

### 4.5 Linking OCT metrics to radiotherapy planning and technique optimization

The results of this study emphasize the importance of OCT technology in early monitoring of changes in the optic disc structure in patients undergoing head and neck radiotherapy. Through OCT imaging, minor structural changes in the optic disc can be detected early, thereby providing clinicians with a basis for early intervention. Early identification of optic disc damage helps to take timely measures to reduce the impact of radiation-related ocular complications on patients’ visual function. For example, adjusting the radiation dose or adopting protective measures can reduce the risk of optic disc damage. In addition, regular OCT examinations can help monitor changes in the structure of the optic disc and detect potential complications in a timely manner, thereby improving patients’ prognosis (20, 21).

The earlier observation in the results section—that the lateral nasal macular region showed the most significant thinning at 6 months post-radiotherapy (P<0.0001)—can be explained by the anatomical characteristics of this region: the lateral nasal macula is closer to the orbital apex, and when radiotherapy fields involve the skull base or sphenoid sinus (as in 45% of patients in our study, **Table 2**), this region receives a higher scattered radiation dose. Additionally, the lateral nasal macular region has a relatively sparse choroidal vascular network compared to the central macula, making it more vulnerable to radiation-induced microcirculation impairment, which aligns with the mechanism of CVI-mediated damage discussed above.

### 4.6 Clinical implications for personalized radiotherapy

Based on the findings of this study, we propose a personalized ocular protection strategy for patients undergoing head and neck radiotherapy, divided into three phases:

Pre-radiotherapy planning constraints: We recommend incorporating “baseline OCT parameters” (especially CVI and lateral nasal macular thickness) into pre-radiotherapy assessments. For patients with baseline CVI < 60% (indicating poor choroidal microcirculatory reserve) or lateral nasal macular thickness < 300 μm, the cumulative dose to ocular OARs should be strictly constrained to ≤4000 cGy; if necessary, radiotherapy fields should be adjusted to avoid the orbital apex region. <mark>For patients with superficial tumor targets (e.g., isolated mandible), where ocular dose is inherently low (Dmean <1600 cGy, V40 = 0%, Section 3.7), dose constraints can be moderately relaxed, and OCT monitoring intervals extended (e.g., every 6–12 months post-radiotherapy).

Intra-radiotherapy adaptive strategies: If a patient exhibits a CVI decrease ≥5% or lateral nasal macular thickness thinning ≥10 μm (early damage signals) during radiotherapy (or at 3 months post-radiotherapy), adaptive adjustment of the radiotherapy plan should be initiated promptly, such as reducing the fractional dose (from 200 cGy/fraction to 180 cGy/fraction) or optimizing field boundaries to reduce scattered radiation dose to the eyes. <mark>For patients with V40 approaching 5% during treatment, “custom lead shielding” (0.5mm lead block on the orbital apex side) or intensity-modulated radiotherapy (IMRT) dose sculpting should be used to limit further high-dose exposure to ocular OARs.

Post-radiotherapy personalized monitoring: An OCT monitoring schedule should be developed based on “radiation dose + radiotherapy field + volume metrics” stratification. <mark>High-risk patients (dose >4000 cGy, V40 >5%, or radiotherapy fields involving the skull base/sphenoid sinus) are recommended to undergo OCT examinations at 3, 6, 9, and 12 months post-radiotherapy; low-risk patients (dose ≤4000 cGy, V40 <5%, or superficial targets) can undergo examinations every 6–12 months until 2 years post-radiotherapy.</mark> This strategy enables closed-loop management of “precision assessment - dynamic adjustment - stratified monitoring,” minimizing radiotherapy-related ocular damage.

Further analysis of radiotherapy field distribution data in this study (**Table 2**) reveals that 45.00% of patients received “mandible combined with most of the maxilla” radiotherapy—a field that often requires coverage of the skull base or sphenoid sinus to control tumors. At 6 months post-radiotherapy, the thinning amplitude of lateral nasal macular thickness in these patients (average reduction of 15.4 μm) was significantly greater than that in patients with radiotherapy fields not involving the skull base (average reduction of 6.2 μm, P = 0.028). <mark>This association is further supported by our dose-volume data: patients with skull base/sphenoid sinus coverage had higher V40 (8.2 ± 3.1% *vs*. 1.5 ± 0.8% in non-skull base group, P<0.001), confirming that scattered radiation from orbital-adjacent fields drives ocular injury. For these patients, IMRT dose sculpting to limit V40 <5% is particularly critical (25).

### 4.7 Limitations and future directions

Although this study provides important data on the impact of head and neck radiotherapy on optic disc structure, there are still some limitations. First, the sample size of this study is relatively small, which may affect the statistical significance of the results. Future studies need to include more patients to further validate the findings of this study. Second, the follow-up period of this study is relatively short and does not fully assess the long-term structural changes in the optic disc after radiotherapy. Notably, this study has initiated an extended follow-up program: the 60 enrolled patients are undergoing OCT and functional endpoint (BCVA, visual field) follow-up at 12 and 24 months post-radiotherapy. Among the 28 patients who have completed the 12-month follow-up, a continuous CVI decrease was observed in the >4000 cGy group (from 59.15% to 58.30%, P = 0.031), while no significant change was seen in the ≤4000 cGy group. Relevant long-term data will be reported in subsequent studies to clarify the pattern of delayed ocular structural damage caused by radiotherapy. Long-term follow-up studies will help better understand the long-term impact of radiotherapy on the optic disc. In addition, this study did not include a control group of radiotherapy patients, and future studies can consider setting up a control group to more accurately assess the impact of radiotherapy on optic disc structure. Finally, this study only focused on changes in optic disc structure, and future studies can further explore the impact of radiotherapy on optic disc function, such as visual field defects and vision loss.

In summary, this study assessed the impact of head and neck radiotherapy on optic disc structure using OCT and found that radiotherapy can lead to significant changes in the structure of the optic disc. These findings provide a new perspective for understanding radiation-related ocular complications and highlight the importance of OCT technology in early monitoring of optic disc damage. Future studies need to further validate these findings and explore the potential impact of radiotherapy on optic disc function.

## 5 Conclusions

In conclusion, our study demonstrates that head and neck RT induces significant changes in optic disc parameters, as evidenced by alterations in RNFL thickness and choroidal vasculature, as assessed by OCT. These findings highlight the importance of OCT as a non-invasive tool for early detection and monitoring of optic disc changes in patients undergoing RT. Early identification of these changes can facilitate timely intervention to mitigate the risk of vision-threatening complications. The personalized strategy proposed in this study—”baseline OCT guiding dose constraints, early OCT changes triggering adaptive adjustments, and risk-stratified monitoring”—provides specific evidence for clinical decision-making in head and neck radiotherapy, promoting a shift from “tumor control priority” to “balance between tumor control and organ protection.” Future research should focus on larger cohorts and longer follow-up periods to further elucidate the long-term impact of RT on optic disc structure and function. Additionally, exploring the potential benefits of prophylactic measures and therapeutic interventions to protect the optic disc during RT may be warranted to improve visual outcomes in this patient population.

## Data Availability

The original contributions presented in the study are included in the article/**Supplementary Material**. Further inquiries can be directed to the corresponding author.
