# Supplementary material for: Assessment of optic disc parameter changes in head and neck cancer patients undergoing radiotherapy based on OCT
Source: Front Oncol. 2026 Jan 12;15:1674684. doi: 10.3389/fonc.2025.1674684 (PMC12833692; doi:10.3389/fonc.2025.1674684)
Supplement: Supplementary file 1 [file Table1.docx]

**Supplementary Table S1 Comparison of OCT Parameters by Radiation Dose Stratification**

| Parameter | Dose Group | Baseline | 6 Months Post-RT | P Value |
| --- | --- | --- | --- | --- |
| Choroidal Vascular Index (CVI, %) | ≤4000 cGy (n=8) | 60.85±3.21 | 59.90±2.98 | 0.412 |
|  | >4000 cGy (n=52) | 60.52±3.70 | 59.15±2.80 | 0.008 |
| Lateral Nasal Macular Thickness (μm) | ≤4000 cGy (n=8) | 311.2±18.7 | 302.7±17.9 | 0.189 |
|  | >4000 cGy (n=52) | 313.8±19.2 | 296.6±20.5 | 0.035 |
| Inner Nasal RNFL Thickness (μm) | ≤4000 cGy (n=8) | 338.5±19.8 | 336.7±20.1 | 0.621 |
|  | >4000 cGy (n=52) | 337.2±20.3 | 333.0±20.5 | 0.049 |
| Note: Data are presented as mean±standard deviation; P values were calculated using the Mann-Whitney U test for non-normally distributed data. | | | | |
